# Montessori Impacts on Long-term Care Staff: Results of a Stepped-Wedge Cluster Randomized Trial

**DOI:** 10.1093/geroni/igaf122.063

**Published:** 2025-12-31

**Authors:** Michelle Hilgeman, Amber Collins, Whitney Gay, Teddy Bishop, Kimberly Curyto, A Lynn Snow, Whitney Mills, Richard Kennedy

**Affiliations:** Tuscaloosa VA Medical Center, Tuscaloosa, Alabama, United States; Tuscaloosa VA Medical Center, Tuscaloosa, Alabama, United States; Tuscaloosa VA Medical Center, Tuscaloosa, Alabama, United States; Tuscaloosa VA Medical Center, Tuscaloosa, Alabama, United States; VA Western New York Health Care System, Batavia, New York, United States; The University of Alabama, Tuscaloosa, Alabama, United States; Brown University, Providence, Rhode Island, United States; The University of Alabama at Birmingham, Birmingham, Alabama, United States

## Abstract

Person-centered care practices and staff outcomes have been associated in cross-sectional studies in long-term care settings. However, few clinical trials have examined changes in staff outcomes following introduction of a person-centered intervention delivered to residents. This study is among the first to examine the impact of Montessori approaches on staff outcomes for an intervention focused on meaningful engagement, strengths identification, empowerment, and dignity support of residents. We conducted a hybrid type 3 effectiveness-implementation study in 8 US Veterans Health Administration Community Living Centers (CLCs, VA nursing homes) using a stepped wedge cluster randomized trial design. Staff completed online surveys of perceptions of implementation success and effectiveness during pre-intervention or control time points (i.e., baseline and 1-month before baseline, N = 229) and post-intervention (i.e., 3-months and 6-months after training, N = 218). Changes were modeled over time using generalized estimating equations. Implementation results indicated significant changes in perceptions about whether Montessori would become/was a normal part of routine care within their CLC (p < .001). Staff-reported effectiveness outcomes reflected post-intervention changes of lower staff burnout scores (p = .028) and higher person-centered care scores (p = .014) on a social connectedness and individualized care and services scale. Collection of survey data from staff about their experiences expands understanding of both implementation and effectiveness outcomes in clinical trials of non-pharmacological interventions. Approaches like Montessori may have the potential to improve both person-centered care practices for residents and outcomes for the long-term care staff workforce.

